# Knowledge, Attitudes, Practices on Antimicrobial Use in Animals Among Livestock Sector Stakeholders in Kenya

**DOI:** 10.1155/2024/8871774

**Published:** 2024-11-19

**Authors:** Jack O. Omolo, Ruth Omani, Mark A. Caudell, Tabitha Kimani, Stella Kiambi, Folorunso O. Fasina

**Affiliations:** ^1^Department of Agriculture, Livestock Development and Blue Economy, County Government of Kilifi, Kilifi 80101, Kenya; ^2^Food and Agriculture Organization of the United Nations—ECTAD, Nairobi 00100, Kenya; ^3^Food and Agriculture Organization of the United Nations—ECTAD Regional Office for Eastern Africa, Nairobi 00100, Kenya; ^4^Food and Agriculture Organization of the United Nations—ECTAD, Dar es Salaam 14110, Tanzania

**Keywords:** animal health service provider, antimicrobial resistance, antimicrobial use, Kenya, livestock

## Abstract

**Background:** Antimicrobials are used on farms to manage livestock diseases. In many developing countries, antimicrobial use (AMU) is insufficiently controlled, and antimicrobials are prone to misuse and abuse, thereby fostering the emergence of antimicrobial resistance (AMR). AMR remains a challenge in Kenya, and the extent remains unknown. This study assessed the knowledge, attitudes, and practices (KAP) regarding AMU among multisectoral stakeholders in Kenya.

**Methods:** The cross-sectional survey was conducted in August 2021 among 381 livestock farmers in Busia, Nakuru, and Isiolo Counties, while 47 animal health service providers (AHSPs) and 32 One Health practitioners (OHPs) were enrolled across Kenya. The data collection tool uploaded on KoBoCollect software was used to collect information on demographics, farming systems, KAP on AMR and AMU, and sources of information. Descriptive statistics were performed. Knowledge was either correct or incorrect, while practices were assigned as desirable or undesirable. Bivariable analysis to assess factors associated with KAP using odds ratio (OR) at 95% confidence level (CL). The Pearson correlation test was conducted to test the correlation between demographic independent variables and farmers' KAP, *p* < 0.05.

**Results:** Most farmers, 234 (61.4%), were young adults between 30 and 49 years old. Additionally, 48.9% of the farmers had less than 5 years of experience in farming. Among the AHSPs, 76.6% were male, with 21 (44.7%) having 2–5 years of experience. All (32) OHPs had over 15 years of experience. Correct knowledge in AMR/AMU was observed in 52.6% of the farmers, 88.2% of AHSPs, and all OHPs. Desirable practices were observed in 133 (34.9%) of farmers, 22 (45.1%) of AHSPs, and 25 (76.4%) of OHPs. Among the farmers, having basic education was associated with correct knowledge (OR 4.07, *p*=0.0007); however, being male (OR 1.584, *p*=0.0456) and having a higher education level (OR 1.582, *p*=0.0165) were associated with desirable practices. There was a significant positive correlation between having correct knowledge and level of education (*p* < 0.0001), years of farming, and correct knowledge (*p* < 0.0001). However, years of farming negatively correlated with the desirable practices (*p* < 0.0001). Farmers' preferred sources of information regarding AMR/AMU were friends 130 (33.9%), farmer meetings/workshops 99 (25.9%), and radio 41 (10.7%). AHSPs obtain information from scientific conferences/trainings (17) (65.4%), workshops (13) (50.0%), and TV and radio (12) (46.2%), while OHSPs mostly get information through college training (14) (58.3%) and workshops (8) (33.3%).

**Conclusion:** Correct knowledge of AMR/AMU did not result in adopting the desirable practices. A better understanding of the socioeconomic aspects of welfare, good livestock production measures, and AMU stewardship will be desired. This study provides a foundation for developing effective antimicrobial stewardship, best farm practices, and intervention programs to reduce inappropriate AMU.

**Public Implication:** Farmers' AHSP practices are likely to promote the emergence of AMR, a health challenge for animals and humans.

## 1. Introduction

Bacterial antimicrobial resistance (AMR) is one of the emerging prominent public and animal health threats of our time that threatens the effective treatment of an ever-increasing range of bacterial infections in both animal and human populations [1]. Major international agencies tasked with promoting public and animal health, including the Food and Agriculture Organization, World Health Organization, World Animal Health Organization, United Nations Environmental Program, and researchers, agree that AMR is a pressing global issue that requires coordinated efforts to address [2–4]. Left unchecked, AMR significantly threatens the achievement of the Sustainable Development Goal (SDG), with the most significant potential impact being on no poverty (SDG 1), zero hunger (SDG 2), health and well-being (SDG 3), clean water and sanitation availability (SDG 6), decent work and economic growth (SDG 8), and reduced inequality (SDG 10) [2, 5, 6]. It is estimated that over 24 million persons will become extremely poor by 2030 due to AMR, with collective economic costs surpassing $120 trillion by 2050 [2, 7, 8]. Even though AMR is a global problem, the socioeconomic burden is and will be disproportionately higher in low-income and middle-income countries such as Kenya unless mitigation efforts are intensified [9–11].

Studies and laboratory records in Kenya indicate an increasing prevalence of AMR bacteria in various parts of the country [12, 13]. However, positive steps toward containment and control of AMR have been undertaken through streamlining coordination and implementation structures to mitigate AMR [12–14]. There is increasing awareness of AMR, coordinated surveillance activities in livestock and human health sectors, and implemented field activities to promote infection prevention and control and reduce unjustified antimicrobial use (AMU) [12–14]. Despite these efforts, the burden of AMR and AMU in Kenya has not been determined comprehensively due to a lack of reliable data [12–15].

Regulations of antimicrobial supply chains for use in animals have yet to be fully implemented in Kenya despite government requirements that agroveterinary shops be staffed with pharmaceutical and animal health technicians who have obtained formal training in animal sciences. The agrovet shop attendants may sell antimicrobials but cannot prescribe them [16]. According to local law, owners of agroveterinary shops establishments may be veterinarians and thereby legally able to prescribe antimicrobials, or they may be paraprofessionals. According to the law, veterinary paraprofessionals are supervised by the veterinary professionals who prescribe the antimicrobials. Private veterinary professionals travel to farms at the request of farmers, where they provide advice and animal treatments or prescribe veterinary drugs. Veterinary professionals typically have professional qualifications specifically enabling them to prescribe veterinary antimicrobials and are governed by the Veterinary Surgeons and Veterinary Paraprofessional Act of the Government of Kenya [16]. Farmers can, however, source their antimicrobials directly from the agroveterinary shops monitored by veterinarians, veterinary paraprofessionals, or shop assistants under the veterinarian's guidance. Sometimes, farmers can obtain their medicines at the livestock markets. In our research, we examined the understanding, cultural beliefs, practices, and habits related to AMR and AMU to gain insights into widespread knowledge, attitudes, motivations, and influences that shape behavior concerning antimicrobials in Kenya.

## 2. Results

### 2.1. Demographic Characteristics of the Respondents

The study reached 381 respondent farmers, 249 (65.3%) males. The predominant age group among the farmers was young adults, specifically those aged between 30 and 49 years, accounting for 234 (61.4%) of the total. The farmers had varied levels of education, with the majority having completed secondary education 121 (31.8%). Most of the farmers had less than five years of experience in farming 186 (48.9%). Of the 47 AHSP respondents, 36 (76.6%) were male. Most respondents were young adults aged 20–29, with 21 (44.7%) having 2–5 years of experience in veterinary-related fields. The survey received responses from 32 OHPs, of which the majority were male, with an age group of 20–29 years and 14 (43.8%) being the majority. The respondents had several years of experience, ranging from less than 1 year to over 15 years (Table 1).

### 2.2. Livestock Farmers

#### 2.2.1. Knowledge and Attitudes

More farmers 108 (28.4%) have heard about antibiotics as compared to antimicrobial 51 (13.4%); however, 32 (9.2%) of those who had heard about antibiotics could correctly differentiate the two. Most farmers, 308 (85.7%), have heard of AMR, while 60 (15.8%) of farmers describe what causes AMR. Knowledge on the drivers of emergence of AMR by farmers was varied as 80 (23.1%) attributed to overuse of antimicrobials, 60 (15.8%) reported bacteria developing resistance, 36 (10.4%) reported medicine losing its potency, and 27 (7.8%) reported failure to complete antibiotic dosages following animal health improvements (Table 2).

**Figure 1:**
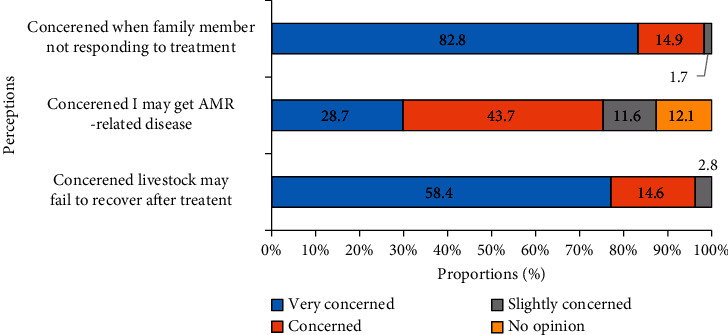
Assessment of farmers' perceptions on antimicrobial resistance (*n* = 355). All responses are in percentages.

**Figure 2:**
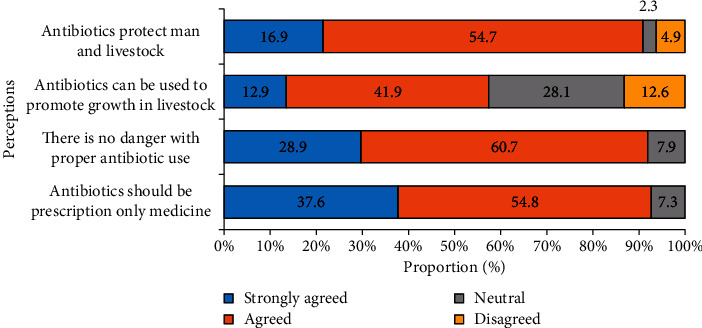
Assessment of farmers' perceptions on antibiotic use (*n* = 355). All responses are in percentages.

Farmers were concerned that their animals and family members would acquire AMR. Failure of an animal to recover from illness was highly regarded as a serious concern. One hundred and forty-nine farmers (41.9%) perceived that it was appropriate to use antimicrobials to promote animal growth although 0.4% claimed to use antibiotics to improve productivity (Figures 1 and 2).

#### 2.2.2. Practices on AMU Among Farmers

Antimicrobials mainly were sourced from agroveterinary shops 179 (49.0%) and veterinary professionals as reported 164 (47.0%). Some farmers, 228 (63.7%), stated that they would obtain a prescription before buying antimicrobials. Likewise, over half of the farmers (60.2%) reported using alternative medicines as the first option instead of antimicrobials. The reason for using alternative medicine was to prevent AMR 145 (86.8%) and their affordability, as reported by 16 respondents (9.6%). Farmers (36.9%) relied on agroveterinary shops' recommendations for quality assurance; checking the expiry dates of antibiotics before purchasing and acquiring antibiotics from the specific outlet were some of the additional quality assurance practices conducted by the respondents. Antimicrobials were used unnecessarily by 121 farmers (34.1%) to treat foot-and-mouth disease and Newcastle disease in chickens by 111 farmers (31.1%). However, 44.5% of farmers kept records of livestock production, and 26.8% kept records of sales at the farm. 16.6% of respondents kept no records (Table 3).

The results of bivariable analysis were conducted to assess the associations between having good knowledge, favorable practices toward AMR and gender, level of education, age group in years, and years of farming experience. Basic education (OR 3.45, CI 2.0155–5.9206) and tertiary education (OR 4.07, CI 2.1246–7.7840) were significantly associated with good knowledge of AMR. There was a weak significant association between gender and having good knowledge of ARM, as the odds of having good knowledge among males were 1.83 times that of females (CI 1.1802–2.8303) (Table 4). When all the independent factors were placed in a multivariate logistic regression, the education level of a farmer (*p* < 0.001) was found to be associated with good knowledge, while gender (*p* < 0.05) and education level (*p* < 0.05) influenced the favorable practices toward AMR (Table 5).

There was a significant positive correlation between having correct knowledge and level of education (*p* < 0.0001), years of farming, and correct knowledge (*p* < 0.01). However, years of farming had a negative correlation with the desirable practices (*p* < 0.01) (Table 5).

### 2.3. Animal Health Service Providers (AHSPs)

#### 2.3.1. Knowledge, Attitudes, and Practices (KAP) Toward AMU and AMR by Animal Health Professionals

Most respondents reported having heard of AMR and could properly describe antibiotics and antimicrobials. The majority had heard about AMR during training programs (17) (65.4%) and in workshops (13) (50.0%) (Table 6). The understanding that AMR is a global threat was agreed to by 22 respondents (84.6%), while 12 respondents (46.2%) responded that AMR can be eradicated. Antimicrobial overuse, as reported by 23 respondents (88.5%), and misuse (73.1%) were the most reported drivers for AMR (Table 6). Practicing hygiene, sanitation, and biosecurity at the farm was agreed as the primary way of preventing the occurrence of AMR by 21 (80.8%) respondents (Table 6).

Most of the respondents reported to have heard of AMR and could properly describe antibiotics and antimicrobials. The majority had heard about AMR during training programs 17 (65.4%) and in workshops 13 (50.0%) (Table 6). The understanding that AMR is a global threat was agreed to by 22 respondents (84.6%), while 12 respondents (46.2%) responded that AMR can be eradicated. Antimicrobial overuse as reported by 23 respondents (88.5%) and misuse (73.1%) were the most reported drivers for AMR (Table 6). Practicing hygiene, sanitation, and biosecurity at the farm was agreed as the main way of preventing the occurrence of AMR by 21 (80.8%) respondents (Table 7).

**Figure 3:**
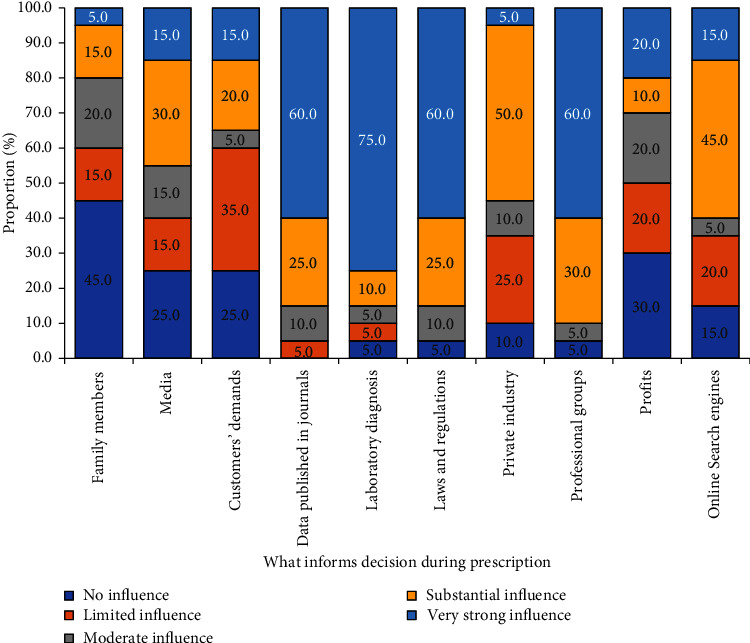
Factors that inform antimicrobial prescription among assessment of animal health service providers (AHSPs).

The respondents shared various perceptions regarding AMU and AMR, with 18 respondents (90.0%) agreeing that antimicrobials improve the growth rate of animals. Over 14 respondents (70.0%) agreed that proper use of antimicrobials in animals poses no danger, while 17 (85.0%) respondents strongly agreed that antimicrobials should be given with prescriptions (Table 4) The respondents identified several factors that strongly influence AMR. These factors included laboratory diagnosis, online journal publications, laws and regulations, and professional groups. According to the data, AHSPs rely mostly on suppliers and distributors for information about specific antimicrobials, including details about dosage and AMR. AHSPs (55.6%) reported relying primarily on suppliers and distributors for information about specific antimicrobials, including dosage and AMR information (Table 8, Figure 3).

Most AHSPs (83.3%) reported advising farmers on antimicrobial use whenever they sell or issue to farmers. Some AHSPs (77.8%) reported that farmers mainly were advised during sales promotion and upon advice request. Few AHSPs (44.4%) demanded prescriptions whenever they sold antimicrobials to farmers. Some AHSPs did not demand prescriptions, citing that it was not required by law (38.9%) or by their organization/business (27.8%). Keeping no records of prescriptions issued and the details on persons presenting the prescriptions was widely practiced by 66.7% of the respondents. Encountering a farmer with expired antimicrobials was a common finding among AHSPs (66.7%), with encounters happening at least once a month (33.3%). Some AHSPs advised farmers to use antimicrobials to treat foot-and-mouth disease in cattle and Newcastle disease in chickens (Table 9).

### 2.4. OHPs

#### 2.4.1. AMU/AMR Knowledge Practices and Perceptions Among OHPs

The OHPs demonstrated satisfactory understanding of AMR and antimicrobial stewardship (see Table 10 for details). The majority of the participants agreed on the beneficial role of antimicrobials in safeguarding both people and animals from diseases. Overall, we observed mostly positive attitudes from OHPs toward AMR. However, more than a quarter strongly agreed on the importance of using antimicrobials in farms. On the concerns of various players in animal health practices and regulations, the respondents were optimistic that the government, farmers, consumers, and feed vendors were slightly concerned about AMR. Respondents representing OH training institutions mostly indicated the inclusion of AMU and AMR-related subjects in the training and research institutions with 72.7% indicating the existence of AMR-related curriculum (Table 11).

## 3. Discussion in This Work

We have evaluated the KAP, of livestock stakeholders on AMU and AMR in Kenya have been evaluated. The profile of evaluated stakeholders spread across levels of education and years of empirical practice. In addition, most of the farmers practice mixed livestock and crop production system. Most farmers in developing countries practice mixed farming in which they rarely specialize in a single production system for their livestock. This method is suitable for small-scale farmers as it spreads the risk of production failures and challenges across various livestock species and crops [17, 18]. Such practices can promote the spread of livestock diseases from one species to another and hinder specialization, which is advocated for increased production [19]. This study observed that the level of education influences good AMR knowledge and practices among farmers. Similarly, there was a direct link between farmers' level of education and their understanding of AMR. This research suggests that both the level of education and farming experience play a significant role in influencing disease prevention practices on farms. Similarly, a number of studies have indicated that education enhances the decision-making abilities of farmers [20, 21]. Furthermore, a study conducted in Ghana noted that farmers with more than 10 years of farming experience were more likely to administer antibiotics to their animals [22]. The practice of treating own animals with antibiotics has been associated with the low number of veterinary professionals against the number of live animals. The inaccessibility of trained professionals and animal treatment costs have been reported as factors that encourage farmers to treat their sick animals [23, 24].

AHSPs were observed to be the least common source of information on AMR to farmers. Most farmers lack access to trained professionals who should inform them of the public health danger of AMR [23, 25]. Despite knowing about the existence of AMR, a few farmers could clearly describe what causes AMR. The proper use of antimicrobials at the farm level has been shown to correlate directly with farmers who are informed on AMR and practice good agricultural practices including adherence to livestock vaccination schedules [16, 25]. Farmers were not observing such practices likely to overuse and abuse antimicrobials, hence promoting AMR [25].

In our study, farmers mostly sourced their antimicrobials from agroveterinary shops and veterinary professionals even as more farmers reported storing expired or left-over medicine for future possible use, a finding that shows poor adherence to good antibiotic use practices [23, 24, 26, 27]. Some farmers reported using alternative herbal medicines, with the aim of reducing the use of antibiotics and emergence of AMR. The application of herbal medicine and other traditional African alternative medicine has shown positive results in treating common illnesses in both livestock and humans [28, 29]. The involvement of alternative medicine can reduce the consumption of antimicrobials, thus decreasing selective pressure for AMR emergence. Nevertheless, it is important to allocate more research efforts toward identifying the active components and the appropriate dosages and investigating the potential side effects of these alternative remedies [30].

Farmers relied on agroveterinary shops' recommendations for antimicrobial administration and quality assurance, and for checking the expiry dates before purchase. Whether this is enough is doubtful, as agroveterinary shops are traders who may be motivated by profits. Quality assurance of antimicrobials used in animals and humans is a critical control point for AMR to assure the potency and efficiency of antimicrobials [31]. Antimicrobials were administered by farmers through injections while mixing with water and other liquids before administration was also a common delivery method. Self-administration of drugs including antibiotics to animals without prescription has been considered one of the main drivers of AMR [32–34]. The use of antimicrobials to treat foot-and-mouth diseases, Newcastle disease in chicken among other viral diseases was practiced (Table 3). Such diseases can easily be managed through adequate vaccination programs, and this will reduce the cost of antimicrobials and the emergence of AMR. Poor farming practices including the lack of adherence to vaccination and biosecurity guidelines, which have been shown to promote the overreliance of antimicrobials [35, 36].

Records-keeping has been used as a tool, which aids in the evaluation of livestock enterprises and assisting in decision-making [37–39]. In our study, records-keeping was low for livestock population, sales at the farm, vaccination, AMU, and disinfection. Almost one-fifth of respondents kept no records. Poor records-keeping behaviors among small-scale farmers can be attributed to low education levels, insufficient awareness, and information [37–39]. Poor record keeping on farms impedes monitoring of farm antimicrobial consumption [40, 41].

Despite 47 veterinary professionals responding, few respondents completed the data collection tool and gave adequate information (63.8%). This was attributed to the data collection method (use of social media or email addresses) to access the respondents rather than physical evaluation. COVID-19 restrictions were still in Kenya. The electronic method was the best system to carry out the survey. Our findings reveal greater familiarity among animal health professionals with terminologies related to antibiotic resistance, a finding reported in a previous study in Kenya [42]. The good knowledge and awareness of AMR by the respondents in this category could be attributed to the various AMR awareness activities carried out in the country that have targeted professionals [14, 26, 43, 44].

The response from farmers on the consult with agroveterinary shops and veterinary professionals is good for antimicrobial stewardship. Animal health professionals are expected to issue prescriptions whenever antimicrobial is indicated to manage livestock diseases [45, 46]. In addition, animal health professionals are routinely and directly involved in AMU on the farm [45–47]. The need for professionals to strictly follow clear guidelines and standardized antimicrobial prescription regulations is obvious [48–50]. The survey demonstrates good knowledge of antimicrobials and AMR among animal health professionals. We relate this to ongoing AMR awareness and that most respondents were young adults who left college recently, as observed in other studies [2, 14, 27].

We observed various practices concerning the use of antibiotics against common livestock diseases caused by viruses. This suggests that vaccines are misused among animal health professionals, or the farmers are not adequately informed. Increasing livestock vaccination has been identified as one of the major actions that will reduce AMU and the resultant burden of AMR [35, 51, 52]. The practice and implementation of the prescription of antimicrobials have been slowly implemented in developing countries including Kenya [27, 48, 53, 54]. Selling of antimicrobials without prescription can be related to weak enforcement of existing regulatory structures, insufficient pharmacovigilance which also often lead to inappropriate sales, and irresponsible acts within the livestock sector in the country. Finally, veterinary professionals have relied on personal experience, peers, laboratory sensitivity test results, ease of administration, and availability of a given type of antimicrobial [27, 55]. Poor or weak regulation makes access to antimicrobials easy for all; hence, AHSPs do not see the need to comply with the need for prescription for antimicrobials.

We observed high knowledge levels of AMR among the OHPs and partners. The majority of the professionals had over 15 years of working experience, had postgraduate educations, had good levels of knowledge of antimicrobials, and practiced stewardship of antimicrobials. Their responses, which were influenced by their perceptions and exposure, were positive in mitigating antimicrobials, and their concerns were apt. These individuals can become facilitators of good practices and training on antimicrobial stewardship among producers and consumers and at various levels of government regulators. The awareness surveys, in addition to contributing to the knowledge of AMR research, aid in increasing awareness of AMR in line with the global goals as outlined in the AMR global action plan. AMR affects animals, humans, and the environment with impacts across the board, hence the need for an all-inclusive approach to handling it, as envisaged in various action plans [14, 27, 43, 53].

This work has its limitations, so it should be carefully interpreted. The study presents the perspectives, knowledge, and attitudes of various groups. Although efforts were made to ensure a diverse sample size, there may be some bias. The work is intricate and condensed, and it is possible that some details and clarity were lost in the complexity of the questions and responses. However, this is a comprehensive effort to compare antimicrobial knowledge, perceptions, and practices among different stakeholders in the livestock industry. It lays the groundwork for refining this knowledge and implementing a more detailed study.

## 4. Materials and Methods

### 4.1. Survey Design

A cross-sectional survey was carried out in the month of August 2021.

### 4.2. Study Site

**Figure 4:**
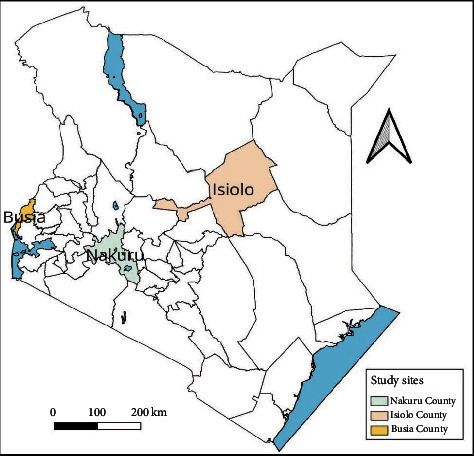
Map of Kenya, showing Nakuru, Busia, and Isiolo Counties where farmer respondents were recruited.

Counties of Busia, Nakuru, and Isiolo were purposively picked to represent small-scale farmers (Busia County), large-scale (Nakuru County), and pastoralists (Isiolo County) (Figure 4). AHSPs and OHPs were enrolled across the country.

### 4.3. Sample Size Determinations

The sample size for farmers was determined using the Cochran formula [56] with an expected favorable attitude and practices set at 50%, 95% confidence level, and a 5% margin of error; hence, a minimum sample size of 384 farmer respondents was targeted. AHSPs were recruited nationwide by sharing the data collection tool through emails and social media platforms including WhatsApp software. OHPs were selected based on their positions and roles at various institutions, including those involved in research, training, diagnostics, government health and animal health departments, and government regulatory bodies. Individuals in charge of One Health activities at these institutions were included in the survey.

### 4.4. Survey Population

The survey was conducted among 381 livestock farmers, 47 AHSPs, and 32 OHPs. Farmers considered for the study are those consenting and must have been practicing livestock farming in the respective county for at least 1 year by the time of the survey. AHSPs are veterinary professionals and paraprofessionals duly registered in Kenya by the Kenya Veterinary Board (KVB) under veterinary surgeons and paraprofessionals act. OHPs were identified from institutions where they conduct One Health-related activities. Such institutions included training institutions, research institutions, and regulatory institutions. Persons heading the One Health activities in these institutions or their assigned replacements were included as respondents in the survey.

### 4.5. Survey Procedure

The sample size for farmers targeted for the survey was divided equally in the three counties giving a minimum sample size of 128 per county. In each county, one subcounty was selected randomly for the survey. Central locations were established in the subcounty with help from the subcounty veterinary officer, which was considered the starting point for the enrollment of the farmer respondents. Using an abstract transect, each subcounty was divided into four quadrants. The first farmer household directly in front of the main entrance was enrolled, and enumerators continued the possible straight line while skipping 10 farmer households until the sample size of 32 per quadrant was reached. Eligible farmers not consenting were replaced with immediate neighbors.

Contacts for AHSPs were obtained from the KVB register published in 2021 where email addresses and phone numbers were captured. The data collection tool was shared with respondents through email addresses and social media platforms including veterinary-based group email platforms, WhatsApp and Facebook social platforms. The targeted respondents were routinely reminded to participate through mail reminders. Contacts and Email addresses of office holders of OHPs were sought, and data collection tools were shared.

### 4.6. Data Collection

A set of questionnaires were developed and validated at the Food and Agriculture Organization of the United Nations (FAO) in Kenya for each category of participants. Pilot testing was done among layer chicken farmers in Kiambu, the locality in the center of Kenya, while the questionnaire for partners was validated through administration among peers in teaching and research institutions. The participants were taken through informed consent before the questionnaire was administered. Data from farmers were collected by trained enumerators drawn from the respective offices of the county director of veterinary services. The enumerators were trained in data collection skills, administration of questionnaire, description of each question, data collection ethics, and consenting. Questionnaire for farmers was administered through a face-to-face interview. Data collection tool for farmers respondents was prepared in KoBoCollect software and uploaded into android smart mobile phones.

Data were collected on demographics—gender, age, level of education, years of experience; KAP, and perceptions on disease management and reporting and views on antimicrobials. Sources of information regarding AMU and resistance were also sought from respondents.

### 4.7. Data Analysis

Data were downloaded into a Microsoft Excel spreadsheet for cleaning and coding (Microsoft Corp, Redmond, Washington, USA). Analysis was done using MS Excel and Epi Info TM V7.2 for Windows [57]. Descriptive statistics were performed by calculating frequencies and proportions in percentages. Knowledge was assigned either correct or incorrect. A set of 10 questions rating the level of knowledge of respondents were issued to include know of AMR, antibiotics and antimicrobials, ability to differentiate between antibiotics and antimicrobials, human and animals share antibiotics, and drivers of AMR. On attitudes, data were collected on indications and use of antimicrobials and concerns on antimicrobial emergence. Practices were assigned as either desirable or undesirable and were assessed through submission of a set of 10 including farm hygiene practices such as biosecurity, provision of antimicrobial supplements in feeds, livestock treatment practices at farms, frequency of AMU, use of prescription for antibiotics, ways of confirming antibiotic quality, disposal of antimicrobials, and dosage completion.

Bivariate and multivariate regression analyses were conducted to assess the level of association between the independent variables of the farmer and the dependent outcomes of knowledge and practices. Multicollinearity analysis was done using the Pearson correlation test to check intercorrelation among the independent variables including age group, sex, education level, and years of farming experience. Associations whose *p* value was < 0.05 were considered statistically significant. In a multivariate logistic regression test, all independent variables were included in the analysis to assess the association with knowledge and practices of AMR among farmers. Resultant associations whose *p* value was < 0.05 were considered statistically significant [58].

### 4.8. Study Limitations

The reliance of the KVB register for the list of AHSPs might not be conclusive as not all AHSPs are registered and retained by the board. However, the register provides legal and list of the majority of AHSPs who are actively practicing in Kenya. The online administration of questionnaire to AHSPs and OHPs provided cheap and convenient way of gathering information. It was noted that low completion rates and low response rates from targeted respondents might have been a consequence of this passive approach. The researchers reached out to the targeted respondents through email and phone call reminders, an action that increased the number of responses. OHSP and OHP were not sensitized on the data collection tool, something which might have affected their way of responding. This group of respondents had minimum tertiary level of education, active practitioners of either animal health or One Health; hence, it was believed that they could read and understand the questions. It was observed that not all respondents completed the questionnaire, resulting in some missing data for subsequent variables.

## 5. Conclusions

In the current research, the majority of farmers rely on guidance from agroveterinary shops when choosing and purchasing antimicrobials or antibiotics. Surprisingly, AHSPs are the least common source of information for farmers on AMR, and many farmers have limited knowledge about AMR. It was found that most farmers and animal health providers do not keep records of the antimicrobials they prescribe or use. The level of education influenced farmers' knowledge of AMR, while both education level and gender influenced practices that contribute to AMR. AHSPs mainly prescribe antimicrobials for treating foot-and-mouth disease in cattle and Newcastle disease in chickens. These diseases are viral and preventable by vaccines.

## Figures and Tables

**Table 1:** Demographic characteristics of the respondents.

Parameters	Farmers (*n* = 381) *f* (%)	Animal health service providers (*n* = 47) *f* (%)	One Health practitioners (*n* = 32) *f* (%)
Gender			
Male	249 (65.3)	36 (76.6)	17 (53.1)
Female	132 (34.7)	11 (23.4)	15 (46.9)
Age groups			
< 20	3 (0.8)	21 (44.7)	0
20–29	76 (20.0)	14 (36.2)	14 (43.8)
30–39	117 (30.7)	7 (14.9)	4 (12.5)
40–49	117 (30.7)	3 (6.4)	9 (28.1)
50–59	54 (14.3)	2 (4.3)	2 (6.3)
≥ 60	14 (3.7)	0	3 (9.4)
Level of education			
None	115 (30.2)	0	0
Primary	65 (17.1)	0	0
Secondary	121 (31.8)	0	0
Tertiary	80 (21.1)	47 (100)	32 (100)
Years of experience in the livestock value chain			
< 1	20 (5.3)	2 (4.3)	6 (18.8)
1–5	166 (43.6)	23 (48.7)	9 (28.1)
6–10	60 (15.8)	8 (17.0)	3 (9.4)
11–15	52 (13.7)	7 (14.9)	5 (15.6)
> 15	83 (21.8)	7 (14.9)	9 (28.1)

**Table 2:** Knowledge on antimicrobial use among farmers.

Parameters	Frequency	%
Awareness of ARM	308	85.7
Know antibiotics	78	20.4
Differentiate antibiotics and antimicrobials	35	9.2
AMR is global threat	45	11.8
AMR cannot be eradicated	369	96.8
AMR affect humans	375	98.4
Aware of drivers of AMR	57	15.0

**Table 3:** Preferred practices regarding antimicrobial use (AMU) among livestock farmers.

Variable	Parameter	*N* (%)
Reason for using antimicrobials (*n* = 235)	Treatment	187 (79.6)
	Advice by veterinarian	25 (10.6)
	Prevent animals from sickness	22 (9.4)
	Increase productivity	1 (0.4)

Why use alternative to antimicrobials (*n* = 209)	Prevent resistance	145 (86.8)
	Available cheap option	16 (9.6)
	Prefer traditional	2 (1.2)
	Veterinarian's advice	4 (2.4)

Source of antimicrobials (*n* = 349)	Agroveterinary shops	171 (49.0)
	Veterinarians	164 (47.0)
	Friends	14 (4.0)

Frequency of antimicrobial use (*n* = 322)	Whenever animal is sick	249 (77.3)
	Weekly	32 (9.9)
	Monthly	33 (10.3)
	Never	4 (1.2)
	Twice a year	4 (1.2)

Usually get prescription (*n* = 355)	Yes	228 (63.7)
	Sometimes	98 (24.6)
	No	29 (8.3)

Source of advice before buying antimicrobials (*n* = 163)	Agroveterinary shops	112 (63.7)
	Local veterinarians	39 (23.9)
	No advice	12 (7.4)

Antimicrobial quality assurance (*n* = 355)	Seller recommendations	131 (36.9)
	Check expiry	195 (54.9)
	Buy from agroveterinary shops	82 (23.1)
	Buy specific brands	58 (16.3)
	Ask friends/neighbor	29 (8.2)
	No quality assurance	6 (1.7)

Antimicrobial administration (*n* = 355)	Through injection	243 (68.5)
	Mix with water/other liquids	151 (42.5)
	Mix with feeds	66 (18.6)

Duration of antimicrobial use (*n* = 355)	As prescribed	220 (62.0)
	As per manufacturer's instruction	84 (23.7)
	Before end of prescription	25 (7.0)
	Longer than prescription	15 (4.2)

Record keeping (*n* = 355)	Livestock population	158 (44.5)
	Sales	106 (29.9)
	Vaccination	95 (26.8)
	Antimicrobials used	49 (13.8)
	Disinfection	23 (6.5)
	No record kept	59 (16.6)

Diseases treated by antimicrobials (*n* = 355)	Treat FMD (viral)	121 (34.1)
	Treat NCD (viral)	111 (31.3)
	Treat mastitis (bacterial)	99 (27.9)
	Treat classical swine fever (viral)	21 (5.9)
	Treat CCPP/Pox (viral)	14 (3.9)

Disposal of excess/expired antimicrobials (*n* = 355)	Keep for future use	244 (68.7)
	Give to neighbors	42 (11.8)
	Throw in garbage	35 (9.9)
	Bury	27 (7.6)
	Burn	16 (4.5)
	Veterinarians take it away	12 (3.4)

*Note:* Note that where the preferred method is selected, it does not prevent other selections to be added as a second or third option.

Abbreviations: CCPP = contagious caprine pleuropneumonia, FMD = foot-and-mouth disease, NCD = Newcastle disease.

**Table 4:** Bivariate analysis of factors associated with farmers' good knowledge and favorable practice*s*.

Parameters		Proportion (%)	Odds ratio (OR)	Confidence interval (CI)
*Good knowledge*				
Gender	Male	53.8	Ref	
	Female	50.8	0.89	0.5798–1.3497

Education level	No formal	58.6	Ref	
	Basic	62.7	3.45	2.0155–5.9206∗
	Tertiary	24.9	4.07	2.1246–7.7840∗

Age in years	20–39	52.9	Ref	
	> 40	51.9	0.64	0.9623–1.4411

Year of experience	< 1	85.0	Ref	
	1-2	50.0	5.6667	1.3341–33.4240∗
	2–5	55.8	4.4826	1.1933–24.0958∗
	5–10	56.8	4.3333	1.0664–25.0958∗
	10–15	50.0	5.6667	1.3639–23.0384∗
	> 15	41.0	8.1667	2.0854–45.9513∗

*Favorable practices*				
Gender	Female	51.4	Ref	
	Male	65.9	1.83	1.1802–2.8303∗

Education level	None	30.2	Ref	
	Basic	66.5	4.5833	2.6699–7.8679∗
	Tertiary	57.5	3.1222	1.6480–5.9150∗

Age group in years	< 20	66.8	Ref	
	20–39	59.1	0.7215	0.0121–9.6376
	> 40	53.5	0.5756	0.0096–11.2660

Year of experience	< 1	70.0	Ref	
	1–2	64.4	1.1290	0.3619–3.5226
	2–5	59.2	1.6103	0.5788–4.4806
	5–10	58.3	1.6667	0.5629–4.9344
	10–15	48.1	2.5200	0.8386–7.5726
	> 15	47.0	2.6325	0.9221–7.5153

^∗^Significant associations.

**Table 5:** Multivariate logistic regression analysis of factors associated with farmers' good knowledge and favorable practice*s*.

Term	Odds ratio	95% UCI	LCI	*p* value
*Good knowledge*				
Farming experience in years	0.8927	0.7382	1.0796	0.2419
Gender	0.7060	0.4509	1.1054	0.1280
Education level	1.9241	1.3166	2.8120	0.0007^1^
Age group in years	1.2246	0.7573	1.9805	0.4087

*Favorable practices*				
Farming experience in years	0.9337	0.7716	1.1299	0.4809
Gender	1.5845	1.0071	2.4927	0.0465^1^
Education level	1.5827	1.0876	2.3033	0.0165^1^
Age group in years	1.0252	0.6341	1.6572	0.9193

Abbreviations: LCI: lower confidence interval; UCI: upper confidence interval.

^1^Significant associations.

**Table 6:** Farmers' preferred animal diseases prevention and treatment methods based on the level of education and years of farming experience.

Characteristics	Level of education			Years of farming experience				
	None (*n* = 115) *f* (%)	Basic (*n* = 186) *f* (%)	Tertiary (*n* = 80) *f* (%)	< 1 (*n* = 20) *f* (%)	1–5 (*n* = 166) *f* (%)	6–10 (*n* = 60) *f* (%)	11–15 (*n* = 52) *f* (%)	> 15 (*n* = 83) *f* (%)
Prevention of diseases at the farm								
Practice farm hygiene	86 (74.8)	118 (63.4)	49 (61.3)	11 (55.0)	115 (69.3)	33 (55.0)	33 (63.5)	61 (73.5)
Use commercial medicine	11 (9.6)	62 (33.3)	18 (22.5)	1 (5.0)	34 (20.5)	25 (41.7)	14 (26.9)	17 (20.5)
Use feed supplements	14 (12.2)	93 (50.0)	51 (63.8)	9 (45.0)	78 (47.0)	26 (43.3)	19 (36.5)	26 (31.3)
Action taken when livestock is unwell								
Call agrovet shop	17 (15.3)	28 (15.8)	20 (27.0)	8 (40.0)	34 (21.9)	7 (11.7)	4 (7.8)	12 (15.0)
Call neighbor/friend	4 (3.6)	2 (1.1)	1 (1.4)	1 (5.0)	3 (1.9)	1 (1.7)	0	2 (2.5)
Call a veterinarian	18 (16.2)	106 (58.6)	33 (44.6)	9 (45.0)	74 (47.7)	29 (48.3)	18 (35.3)	27 (33.8)
Treatment of own animals	71 (64.0)	43 (23.8)	19 (25.7)	2 (10.0)	42 (27.1)	23 (38.3)	27 (53.0)	39 (48.8)
Take no action	1 (0.9)	2 (1.1)	1 (1.4)	0	2 (1.3)	0	2 (3.9)	0

*Note:* Cumulative percentage may exceed 100% where multiple responses were selected. Note that where the preferred method is selected, it does not prevent other selections to be added as a second or third option.

**Table 7:** Assessment of animal health service provider (AHSP) knowledge of AMR and AMU.

Variable	Parameter	Number (%)
Knowledge and awareness of antimicrobials	Know and describe antibiotics correctly (*n* = 29)	28 (96.6)
	Know and describe antimicrobials correctly (*n* = 30)	28 (93.3)
	Know the difference between antibiotic and antimicrobial (*n* = 20)	20 (69.0)
	Have heard of antimicrobial resistance (*n* = 28)	28 (100.0)

Source of information about AMR (*n* = 25)	TV	6 (23.1)
	Workshop	13 (50.0)
	Training program	17 (65.4)
	Social media	6 (23.1)
	Radio	3 (11.5)
	Newspapers	8 (30.8)
	Friends/relatives	4 (15.4)

What best describes antimicrobial resistance (*n* = 25)	No treatment due to ineffective medicine	11 (42.3)
	When micro-organisms become irresponsive/less responsive to antimicrobial drugs	21 (80.8)
	Lack of treatment because medicine loses potency and effectiveness	10 (38.5)
	When micro-organisms change	19 (73.1)

Understanding and perception of AMR (*n* = 25)	AMR in food animals is detected by a number of antimicrobial residues in meat	8 (30.8)
	AMR is a global health threat	22 (84.6)
	AMR can be eradicated	12 (46.2)

What promotes AMR (*n* = 25)	Incomplete dosage during antimicrobial treatment	18 (69.2)
	Appropriate use of antimicrobials in people and animals	2 (7.7)
	Using water contaminated with fertilizer, feces, and antimicrobial residues	9 (34.6)
	Unnecessary antimicrobial use in humans	19 (73.1)
	Antimicrobial overuse in animals	23 (88.5)

Ways to prevent AMR (*n* = 20)	Good vaccination programs	18 (69.2)
	Good husbandry practices	19 (73.1)
	Prudent antimicrobial use	19 (73.1)
	Practicing hygiene, sanitation, and biosecurity	21 (80.8)

**Table 8:** Perceptions and concerns of assessment of animal health service providers (AHSPs) regarding antimicrobial resistance.

Variable	Parameter	Frequency (%)
Antimicrobials protect man and animals from diseases (*n* = 20)	Agreed	9 (45.0)
	Disagreed	9 (45.0)

Antimicrobials improve animal growth rate (*n* = 20)	Strongly disagreed	18 (90.0)
	Agreed	1 (5.0)

No danger if antimicrobials are properly used in animals (*n* = 20)	Strongly agreed	14 (70.0)
	Agreed	5 (25.0)

Antimicrobials should be given with prescriptions (*n* = 20)	Strongly agreed	17 (85.0)
	Agreed	3 (15.0)

Concerned that a family member may get illness does not respond to medication (*n* = 20)	Seriously concerned	17 (85.0)
	Concerned	2 (10.0)

Worried of AMR-related issues in the future (*n* = 20)	Seriously concerned	16 (80.0)
	Concerned	4 (20.0)

Government concerned about antimicrobial resistance (*n* = 20)	Concerned	17 (85.0)
	Not concerned at all	2 (10.0)
	No opinion	1 (5.0)

Farmers concerned about antimicrobial resistance (*n* = 20)	Concerned	14 (70.0)
	Not concerned at all	5 (25.0)
	No opinion	1 (5.0)

Consumers concerned about antimicrobial resistance (*n* = 20)	Concerned	16 (80.0)
	Not concerned at all	3 (15.0)
	No opinion	1 (5.0)

**Table 9:** Antimicrobial dispensation practices among AHSPs.

Variable	Parameter	*F* (%)
Source of antimicrobial information (*n* = 18)	Another animal feed store/animal pharmaceutical store	8 (44.4)
	Facts on antimicrobial/antibiotic resistance	3 (16.7)
	From suppliers/distributor/representative	10 (55.6)
	Sales guidance for antimicrobials/antibiotics	4 (22.2)
	The dosage and use in feed and water	10 (55.6)

Selling feed mixed with antibiotics (*n* = 19)	Never	17 (89.5)
	No (done previously but not at the moment)	2 (10.5)

Advice farmers on antimicrobial use (*n* = 18)	Sometimes	1 (5.6)
	Yes	15 (83.3)
	During sale promotions	2 (11.1)

Occasion when farmers are advised on antimicrobial use (*n* = 18)	During sale promotions	2 (11.1)
	In possession of a prescription	12 (77.8)
	Upon request by customer	3 (16.7)

Demand prescription before selling antimicrobials (*n* = 18)	No	5 (27.8)
	Sometimes	5 (27.8)
	Yes	8 (44.4)

Record keeping on antimicrobials used (*n* = 18)	Feel no need	3 (16.7)
	No records of clients with prescriptions kept	11 (61.1)
	No records of prescriptions kept	12 (66.7)
	Not a requirement by organization/business	5 (27.8)
	Not required by law	7 (38.9)

Frequency of meeting farmers who use expired antimicrobial (*n* = 18)	At least once a month	6 (33.3)
	At least once a week	2 (11.1)
	At least once every 6 months	7 (38.9)
	At least once every 2 weeks	2 (11.1)
	Everyday	1 (5.6)

Advice to farmers on antimicrobial disposal (*n* = 18)	Burn	5 (27.8)
	Bury in the ground	3 (16.7)
	Keep for future use	5 (27.8)
	Throw in the garbage	3 (16.7)
	Return to the suppliers	3 (16.7)

Advice on use of antimicrobials (*n* = 18)^∗^	Advise use of antimicrobials to treat FMD	10 (55.6)^∗^
	Advise use of antimicrobials to treat NCD	10 (55.6)

Abbreviations: FMD = foot-and-mouth disease, NCD = Newcastle disease.

^∗^One respondent gave two responses.

**Table 10:** Pearson correlation test between demographic independent variables and farmers' good knowledge and favorable practice*s*.

Good knowledge					
Pearson correlation	Good knowledge	Gender	Education level	Years of farming	Age group
Good knowledge	1.000	−0.029	0.224	−0.145	−0.031
Gender		1.000	0.168	−0.225	−0.076
Education level			1.000	−0.524	−0.210
Years of farming				1.000	0.532
Age group					1.000
Sig. (*p* value)					
Good knowledge	.	0.285	0.000	0.002	0.275
Gender		.	0.001	0.000	0.070
Education level			.	0.000	0.000
Years of farming				.	0.000
Age group					.

**Favorable practices**					
**Pearson correlation**	**Favorable practices**	**Gender**	**Education level**	**Years of farming**	**Age group**

Favorable practices	1.000	0.139	0.188	−0.146	−0.059
Gender		1.000	0.168	−0.225	−0.076
Education level			1.000	−0.524	−0.210
Years of farming				1.000	0.532
Age group					1.000
Sig. (*p* value)					
Favorable practices	.	0.003	0.000	0.002	0.127
Gender		.	0.001	0.000	0.070
Education level			.	0.000	0.000
Years of farming				.	0.000
Age group					.

**Table 11:** Knowledge assessment of AMR among One Health practitioners (OHPs).

Variable	Parameter	Frequency (%)
Knowledge of AMR terminologies (*n* = 24)	Know and describe antimicrobials correctly	23 (95.8)
	Know and describe antibiotics correctly	23 (95.8)
	Differentiated antimicrobials from antibiotics	17 (70.8)

Heard of AMR (*n* = 24)	Friends	7 (29.2)
	Workshops	8 (33.3)
	Radio	2 (8.3)
	TV	6 (25.0)
	Print media	5 (20.8)
	School training	14 (58.3)
	Social media	4 (16.7)

Described aspects of AMR (*n* = 24)	Bacterial resistance	18 (75.0)
	Genetic transfer	12 (50.0)
	When medicine loses potency	12 (50.0)
	Overuse/misuse of drugs	20 (83.3)
	Health issues affecting animals and plants	18 (75.0)

Promoters of AMR (*n* = 24)	Overuse of antimicrobials	20 (83.3)
	Fertilizer and feces in water	13 (54.2)
	Unnecessary use	22 (91.7)
	Incomplete dosage	17 (70.8)

Preventive measures (*n* = 24)	Good agricultural practices	16 (66.7)
	Prudent use of antimicrobials	21 (87.5)
	Vaccinating livestock	18 (75.0)

## Data Availability

The data that support the findings of this study are available on request from the corresponding author. The data are not publicly available due to privacy or ethical restrictions.

## Supporting Information

Data collection tool (Questionnaires for livestock farmers, animal health service providers, and One Health practitioners).

## References

[1] Prestinaci F., Pezzotti P., Pantosti A. (2015). Antimicrobial Resistance: A Global Multifaceted Phenomenon. *Pathogens and Global Health*.

[2] Joshi M. P., Hafner T., Twesigye G. (2021). Strengthening Multisectoral Coordination on Antimicrobial Resistance: A Landscape Analysis of Efforts in 11 Countries. *Journal of Pharmaceutical Policy and Practice*.

[3] Murray C. J., Ikuta K. S., Sharara F. (2022). Global Burden of Bacterial Antimicrobial Resistance in 2019: A Systematic Analysis. *The Lancet*.

[4] Wernli D., Harbarth S., Levrat N., Pittet D. A. (2022). ‘Whole of United Nations Approach’ to Tackle Antimicrobial Resistance? A Mapping of the Mandate and Activities of International Organizations. *BMJ Global Health*.

[5] Gajdács M., Urbán E., Stájer A., Baráth Z. (2021). Antimicrobial Resistance in the Context of the Sustainable Development Goals: A Brief Review. *European Journal of Investigation in Health Psychology and Education*.

[6] Jasovský D., Littmann J., Zorzet A., Cars O. (2016). Antimicrobial Resistance-A Threat to the World’s Sustainable Development. *Upsala Journal of Medical Sciences*.

[7] Dadgostar P. (2019). Antimicrobial Resistance: Implications and Costs. *Infection and Drug Resistance*.

[8] Founou L. L., Founou R. C., Essack S. Y. (2021). Antimicrobial Resistance in the Farm-To-Plate Continuum: More Than a Food Safety Issue. *Future Science OA*.

[9] Iskandar K., Molinier L., Hallit S. (2021). Surveillance of Antimicrobial Resistance in Low- and Middle-Income Countries: A Scattered Picture. *Antimicrobial Resistance and Infection Control*.

[10] Pokharel S., Raut S., Adhikari B. (2019). Tackling Antimicrobial Resistance in Low-Income and Middle-Income Countries. *BMJ Global Health*.

[11] Sulis G., Sayood S., Gandra S. (2022). Antimicrobial Resistance in Low- and Middle-Income Countries: Current Status and Future Directions. *Expert Review of Anti-infective Therapy*.

[12] Bett B. (2018). Situation Analysis on Antimicrobial Resistance Surveillance and Control in Kenya Antimicrobial Resistance. https://amr.cgiar.org/case-study/situation-analysis-antimicrobial-resistance-surveillance-and-control-kenya.

[13] Maina M., Mwaniki P., Odira E. (2020). Antibiotic Use in Kenyan Public Hospitals: Prevalence, Appropriateness and Link to Guideline Availability. *International Journal of Infectious Diseases*.

[14] Wesangula E. N., Githii S., Ndegwa L. (2020). Implementing the National Action Plan on Antimicrobial Resistance in Kenya: Global Expectations, National Realities. *International Journal of Infectious Diseases*.

[15] Cox J. A., Vlieghe E., Mendelson M. (2017). Antibiotic Stewardship in Low- and Middle-Income Countries: The Same but Different?. *Clinical Microbiology and Infection*.

[16] Kemp S. A., Pinchbeck G. L., Fevre E. M., Williams N. J. (2021). A Cross-Sectional Survey of the Knowledge, Attitudes, and Practices of Antimicrobial Users and Providers in an Area of High-Density Livestock-Human Population in Western Kenya. *Frontiers in Veterinary Science*.

[17] Herrero M., Grace D., Njuki J. (2013). The Roles of Livestock in Developing Countries. *Animal*.

[18] Thornton P. K., Rosenstock T., Förch W., Lipper L., McCarthy N., Zilberman D., Asfaw S., Branca G. (2018). A Qualitative Evaluation of CSA Options in Mixed Crop-Livestock Systems in Developing Countries. *Climate Smart Agriculture: Building Resilience to Climate Change, Natural Resource Management and Policy*.

[19] Salaheen S., Chowdhury N., Hanning I., Biswas D. (2015). Zoonotic Bacterial Pathogens and Mixed Crop-Livestock Farming. *Poultry Science*.

[20] Ninh L. K. (2020). Economic Role of Education in Agriculture: Evidence From Rural Vietnam. *Journal of Economics and Development*.

[21] Paltasingh K. R., Goyari P. (2018). Impact of Farmer Education on Farm Productivity Under Varying Technologies: Case of Paddy Growers in India. *Agricultural and Food Economics*.

[22] Phares C. A., Danquah A., Atiah K., Agyei F. K., Michael O.-T. (2020). Antibiotics Utilization and Farmers’ Knowledge of Its Effects on Soil Ecosystem in the Coastal Drylands of Ghana. *PLoS One*.

[23] Caudell M. A., Dorado-Garcia A., Eckford S. (2020). Towards a Bottom-Up Understanding of Antimicrobial Use and Resistance on the Farm: A Knowledge, Attitudes, and Practices Survey Across Livestock Systems in Five African Countries. *PLoS One*.

[24] Mouiche M. M. M., Moffo F., Betsama J. D. B. (2020). Challenges of Antimicrobial Consumption Surveillance in Food-Producing Animals in Sub-Saharan African Countries: Patterns of Antimicrobials Imported in Cameroon from 2014 to 2019. *Journal of Global Antimicrobial Resistance*.

[25] Afakye K., Kiambi S., Koka E. (2020). The Impacts of Animal Health Service Providers on Antimicrobial Use Attitudes and Practices: An Examination of Poultry Layer Farmers in Ghana and Kenya. *Antibiotics*.

[26] Adekanye U. O., Ekiri A., Galipó E. (2020). Knowledge, Attitudes and Practices of Veterinarians Towards Antimicrobial Resistance and Stewardship in Nigeria. *Antibiotics*.

[27] Mangesho P., Caudell M., Mwakapeje E. R. (2021). Knowing Is Not Enough: A Mixed-Methods Study of Antimicrobial Resistance Knowledge, Attitudes, and Practises Among Maasai Pastoralists. *Frontiers in Veterinary Science*.

[28] Aziz M. A., Khan A. H., Adnan M., Ullah H. (2018). Traditional Uses of Medicinal Plants Used by Indigenous Communities for Veterinary Practices at Bajaur Agency, Pakistan. *Journal of Ethnobiology and Ethnomedicine*.

[29] Jayakumar S., Baskaran N., Arumugam R., Sathiskumar S., Pugazhenthi M. (2018). Herbal Medicine as a Live Practice for Treating Livestock Ailments by Indigenous People: A Case Study from the Konar Community of Tamil Nadu. *South African Journal of Botany*.

[30] Howland O. (2021). Patterns of Use, Gathering, Processing and Administration of Herbal and Alternative Medicines Among People and Livestock in Kenya: A Study of Local Knowledge for One Health. *Journal of Global Health Reports*.

[31] Luu Quynh H., Nguyen Thi Bich T., Ta Hoang L., Erickson V. I., Padungtod P. (2021). Quality Testing of Veterinary Antimicrobial Products Used for Livestock in Vietnam, 2018–2019. *PLoS One*.

[32] Alhaji N. B., Isola T. O. (2018). Antimicrobial Usage by Pastoralists in Food Animals in North-central Nigeria: The Associated Socio-Cultural Drivers for Antimicrobials Misuse and Public Health Implications. *One Health*.

[33] Azabo R., Mshana S., Matee M., Kimera S. I. (2022). Antimicrobial Usage in Cattle and Poultry Production in Dar Es Salaam, Tanzania: Pattern and Quantity. *BMC Veterinary Research*.

[34] Gemeda B. A., Amenu K., Magnusson U. (2020). Antimicrobial Use in Extensive Smallholder Livestock Farming Systems in Ethiopia: Knowledge, Attitudes, and Practices of Livestock Keepers. *Frontiers in Veterinary Science*.

[35] Hoelzer K., Bielke L., Blake D. P. (2018). Vaccines as Alternatives to Antibiotics for Food Producing Animals. Part 1: Challenges and Needs. *Veterinary Research*.

[36] Raasch S., Postma M., Dewulf J., Stärk K. D. C., Grosse Beilage E. (2018). Association Between Antimicrobial Usage, Biosecurity Measures as Well as Farm Performance in German Farrow-To-Finish Farms. *Porcine Health Management*.

[37] Gichohi P. (2019). The Role of Record Keeping and Maintenance in Enhancing Decision Making Among Smallholder Dairy Farmers in Gitugi Ward in Murang’a County, Kenya. *Information Development*.

[38] Kuteesa A., Kyotalimye M. (2019). Documentation and Data Handling: How Can Africa Promote Record Keeping and Investment in Data Management?. *African Journal of Food, Agriculture, Nutrition and Development*.

[39] Tham-Agyekum E., Appiah P., Nimoh F. (2010). Assessing Farm Record Keeping Behaviour Among Small-Scale Poultry Farmers in the Ga East Municipality. *Journal of Agricultural Science*.

[40] Cuong N. V., Phu D. H., Van N. T. B. (2019). High-Resolution Monitoring of Antimicrobial Consumption in Vietnamese Small-Scale Chicken Farms Highlights Discrepancies between Study Metrics. *Frontiers in Veterinary Science*.

[41] Schar D., Sommanustweechai A., Laxminarayan R., Tangcharoensathien V. (2018). Surveillance of Antimicrobial Consumption in Animal Production Sectors of Low- and Middle-Income Countries: Optimizing Use and Addressing Antimicrobial Resistance. *PLoS Medicine*.

[42] Muloi D., Fèvre E. M., Bettridge J. (2019). A Cross-Sectional Survey of Practices and Knowledge Among Antibiotic Retailers in Nairobi, Kenya. *Journal of Global Health*.

[43] Fasina F. O., LeRoux-Pullen L., Smith P. (2020). Knowledge, Attitudes, and Perceptions Associated With Antimicrobial Stewardship Among Veterinary Students: A Multi-Country Survey From Nigeria, South Africa, and Sudan. *Frontiers in Public Health*.

[44] Rahman M. S., Rafa N. (2021). Common Barriers, Attitudes, and Practices of Veterinary Practitioners Regarding Antimicrobial Resistance and Stewardship in Chattogram, Bangladesh. *Open Veterinary Science*.

[45] Odoi A., Samuels R., Carter C. N., Smith J. (2021). Antibiotic Prescription Practices and Opinions Regarding Antimicrobial Resistance Among Veterinarians in Kentucky, USA. *PLoS One*.

[46] Tebug S. F., Mouiche M. M. M., Abia W. A. (2021). Antimicrobial Use and Practices by Animal Health Professionals in 20 Sub-Saharan African Countries. *Preventive Veterinary Medicine*.

[47] Aworh M. K., Kwaga J. K. P., Okolocha E. C. (2021). Assessing Knowledge, Attitude, and Practices of Veterinarians towards Antimicrobial Use and Stewardship as Drivers of Inappropriate Use in Abuja, Nigeria. *One Health Outlook*.

[48] Chema S., Gathuma J. M. (2004). Kenya: The Development of Private Services and the Role of the Kenya Veterinary Association. *Revue Scientifique et Technique de l OIE*.

[49] Heffernan C., Misturelli F. (2002). The Delivery of Veterinary Services to the Poor: Preliminary Findings from Kenya. https://assets.publishing.service.gov.uk/.media/57a08d2aed915d3cfd001868/R7359a.pdf.

[50] Jaime G., Hobeika A., Figuié M. (2021). Access to Veterinary Drugs in Sub-Saharan Africa: Roadblocks and Current Solutions. *Frontiers in Veterinary Science*.

[51] Micoli F., Bagnoli F., Rappuoli R., Serruto D. (2021). The Role of Vaccines in Combatting Antimicrobial Resistance. *Nature Reviews Microbiology*.

[52] Vekemans J., Hasso-Agopsowicz M., Kang G. (2021). Leveraging Vaccines to Reduce Antibiotic Use and Prevent Antimicrobial Resistance: A World Health Organization Action Framework. *Clinical Infectious Diseases*.

[53] ReAct AMR Stakeholder Mapping From ReAct Europe – Action on Antibiotic Resistance.

[54] Meseko C., Olabisi M., Ehizibolo D., Muraina I. (2019). Veterinary Pharmaceuticals and Antimicrobial Resistance in Developing Countries. *Veterinary Medicine and Pharmaceuticals*.

[55] De Briyne N., Atkinson J., Pokludová L., Borriello S. P., Price S. (2013). Factors Influencing Antibiotic Prescribing Habits and Use of Sensitivity Testing Amongst Veterinarians in Europe. *The Veterinary Record*.

[56] Lawrie N. L. (1978). *Sampling Technique*.

[57] Centers for Disease Control and Prevention Epi Info TM V7.2 for Windows. https://www.cdc.gov/epiinfo/pc.html.

[58] Denis D. J. (2021). Applied Univariate, Bivariate, and Multivariate Statistics: Understanding Statistics for Social and Natural Scientists, With Applications in SPSS. WILAY Online Library.

