# Supplementary material for: Knowledge, Attitudes, Practices on Antimicrobial Use in Animals Among Livestock Sector Stakeholders in Kenya
Source: Vet Med Int. 2024 Nov 19;2024:8871774. doi: 10.1155/2024/8871774 (PMC11599476; doi:10.1155/2024/8871774)
Supplement: Supporting Information — Additional supporting information can be found online in the Supporting Information section. [file 8871774.f1.docx]

**Knowledge, Attitudes, Practices on Antimicrobial Use in Animals among Livestock Sector Stakeholders in Kenya**

**Data collection tool**

**Farmers Questionnaire**

| no |  |  |
| --- | --- | --- |
| 1 | Respondent ID |  |
| 2 | Collector ID |  |
| 3 | Date |  |
| 4 | County |  |
| 5 | Sub county |  |
| 6 | Ward |  |
| 7 | Email Address |  |
| 8 | Name |  |
| 9 | Gender |  |
| 10 | Age |  |
| 11 | What is your highest education level attained? |  |
| 12 | Country of Residence/Nationality |  |
| 13 | Do you consider farming your main occupation? |  |
|  | If no, what is your other occupation other than farming? |  |
|  | If yes, for how many years have you been farming? |  |
| 14 | What animals (terrestrial or aquatic) do you raise in your farm, what species/breeds and how many? (Select all that apply) | Cattle: |
|  |  | Buffalo: |
|  |  | Goat: |
|  |  | Sheep |
|  |  | Pig: |
|  |  | Chicken: |
|  |  | Fish: |
|  |  | Prawn: |
|  |  | Others (Please specify) |
| 15 | What crops do you grow in different seasons and under what acreage? | Open-Ended Response |
| 16 | Which animal disease outbreak(s) have you encountered or experienced in the last 12 months? (Select all that apply) | Highly Pathogenic Avian Influenza/Bird flu |
|  |  | Foot and Mouth Disease (FMD) |
|  |  | Classical swine fever/Swine Cholera |
|  |  | Swine Flu |
|  |  | Newcastle Disease |
|  |  | Mastitis |
|  |  | No animal disease outbreaks |
|  |  | Others (Please specify name or symptoms) |
| 17 | If you also farm crops, have you encountered or experienced crop disease outbreaks in the last 12 months? |  |
|  | If yes, what diseases? What were the symptoms? |  |
| 18 | What do you do to keep your animals (terrestrial or aquatic)/plants healthy? (Select all that apply) | I leave them alone |
|  |  | I try to keep the farm clean |
|  |  | I give them a special medicine |
|  |  | I give them nutritional supplements |
|  |  | Others (Please specify) |
| 19 | Why do you do what you have answered in Question above? | Open-Ended Response |
| 20 | What do you do FIRST when your animals (terrestrial or aquatic)/plants show sign of sickness? | I call the local veterinarian |
|  |  | I treat them with medicine |
|  |  | I call the agrovet |
|  |  | Others (Please specify) |
|  |  | I leave them alone |
| 21 | Why do you do what you have answered in Question 20? |  |
| 22 | In case you answered that you give them medicine in Questions above, what kind of medicine do you give them? |  |
| 23 | Can you describe what antimicrobials are? If yes, what do you know about it? | Do not know |
|  |  | Yes |
|  |  | No |
| 24 | Can you describe what antibiotics are?If yes, what do you know about it? | Do not know |
|  |  | Yes |
|  |  | No |
| 25 | Do you think there is a difference between antibiotics and antimicrobials? | Do not know |
|  |  | Yes |
|  |  | No |
| 26 | Please list three antimicrobials/antibiotics that you know (generic or brand name) |  |
| 27 | All antimicrobials/antibiotics used in humans are also used in animals, and all antimicrobials/antibiotics used in animals are also used in humans | False |
|  |  | True |
|  |  | I don’t know |
| 28 | Have you heard of antimicrobial resistance or antibiotic resistance? If yes, what do you know about it? | If yes, what do you know about it? |
| 29 | Where did you hear about antimicrobial/antibiotic resistance? (Select all that apply) | From television |
|  |  | From radio |
|  |  | From print media – newspapers, magazines, etc. |
|  |  | From my friends |
|  |  | From my colleagues |
|  |  | From a seminar or workshop |
|  |  | Social media such as Facebook, Twitter or Instagram |
|  |  | School or training program |
|  |  | Other (Please specify) |
| 30 | Select what best describes antimicrobial/antibiotic resistance in the list below. (Select all that apply) | Antimicrobial/antibiotic resistance is dangerous but I do not know how to describe it |
|  |  | When an infection cannot be treated because the medicine is ineffective |
|  |  | When bacteria develop resistance to one or more antimicrobials/antibiotics |
|  |  | I am not sure or I do not know |
| 31 | Which of the following is true regarding the nature of antimicrobial/antibiotic resistance? (Select all that apply) |  |
|  |  | Antimicrobial/antibiotic resistance is when infections cannot be treated because medicines lose their potency or effectiveness |
|  |  | Antimicrobial/antibiotic resistance is when bacteria develop resistance to antimicrobials such as antibiotics |
|  |  | Antimicrobial/antibiotic resistance emergence is accelerated with overuse and misuse of antimicrobials/antibiotics |
|  |  | Antimicrobial/antibiotic resistance occurs when microorganisms change in response to the use of antimicrobials/antibiotics |
|  |  | Antimicrobial/antibiotic resistance traits (genetic elements) can be transferred from one organism to another |
| 32 | Which of the following is true about antimicrobial/antibiotic resistance as a health issue? (Select all that apply) | Antimicrobial/antibiotic resistance is a health issue concerning animals (terrestrial and aquatic), plants and humans |
|  |  | Antimicrobial/antibiotic resistance occurs in human pathogens only |
|  |  | Antimicrobial/antibiotic resistance in food-producing animals is detected by determining the amount of antimicrobial/antibiotic residue in meat samples |
|  |  | Antimicrobial/antibiotic resistance is a global health threat |
|  |  | Antimicrobial/antibiotic resistance can be eradicated |
| 33 | From the list below, please select what you think is appropriate for use of antimicrobials/antibiotics (Select all that apply) | For treatment of infections in animals (terrestrial and aquatic) and crops |
|  |  | To promote growth in animals (terrestrial and aquatic) |
|  |  | To prevent infections in animals (terrestrial and aquatic) and crops |
|  |  | Do not know |
| 34 | Which of the following practices can contribute to the emergence and spread of antimicrobial/antibiotic resistance? (Select all that apply) | Use of antimicrobials/antibiotics in humans when it is not necessary |
|  |  | Overuse of antimicrobials/antibiotics in animals |
|  |  | Discontinuing antimicrobial/antibiotic use once the patient (animal or human) shows improvement |
|  |  | Practicing appropriate use of antimicrobials/antibiotics in animals |
|  |  | Practicing appropriate use of antimicrobials/antibiotics in humans |
|  |  | Using fertilizer or water containing animal feces with antimicrobial/antibiotic residues |
| 35 | Which of the following practices can help control/prevent the development of antimicrobial/antibiotic resistance? (Select all that apply) | Good vaccination programs |
|  |  | Good husbandry practices |
|  |  | Use of antimicrobials/antibiotics as growth promoters |
|  |  | Good Farm hygiene, sanitation, biosecurity |
|  |  | Prudent use of antimicrobials/antibiotics |
|  |  | None of the above |
| 36 | Please describe how the use of antimicrobials/antibiotics could affect you and your family | Open-Ended Response |
| 37 | How would you rate your situation if one of your family members had an infection that cannot be treated with medicines? | Response |
| 38 | Are you concerned you will get an antimicrobial/antibiotic resistance related disease? | Response |
| 39 | How would you rate your situation if one of your animals had an infection that cannot be treated with medicines? | Response |
| 40 | Please rate your opinion on the following statements whether you Strongly agree; Agree; are Neutral; Disagree; or Strongly disagree: | Antimicrobials such as antibiotics protect both humans and animals from diseases |
|  |  | It is appropriate to use antimicrobials/antibiotics to improve growth of animals |
|  |  | There is no danger in giving antimicrobials/antibiotics to humans if properly used when required |
|  |  | There is no danger in giving antimicrobials/antibiotics to animals if properly used when required |
|  |  | Antimicrobials/antibiotics should be given with prescription |
|  |  | It is important to use antimicrobials/antibiotics in farms |
| 41 | Please rate your interest in learning more about antimicrobials/antibiotics | Response |
| 42 | Do you use antimicrobials/antibiotics in your farm? | Response |
|  |  | If yes when and for what? |
|  | If you are using antimicrobials/antibiotics in your farm, would you be willing to use an alternative instead of antimicrobials/antibiotics? | Response |
|  |  | Why? |
|  | Why do you use antimicrobials/antibiotics in your farm? (Select all that apply) | For treatment of infections in animals (terrestrial and aquatic) and crops |
|  |  | To promote growth in animals (terrestrial and aquatic) |
|  |  | To prevent infections in animals (terrestrial and aquatic) and crops |
|  |  | Others (Please specify) |
| 43 | Where do you get the antimicrobials/antibiotics you use in your farm? (Select all that apply) | From friends |
|  |  | From veterinarian |
|  |  | Animal feed store |
|  |  | Animal pharmaceutical store |
|  |  | Human pharmacy |
|  |  | Others (Please specify) |
| 44 | How often do you get the antimicrobials/antibiotics you use in your farm? | Open-Ended Response |
| 45 | What is the size of your farm? | Response |
| 46 | How often do you use antimicrobials/antibiotics in your farm? | Response |
| 47 | Do you usually get a prescription or professional advice before buying antimicrobials/antibiotics for use in your farm? | Response |
| 48 | Why do you not seek professional advice in buying antimicrobials/antibiotics? (Select all that apply) | It is easy to buy directly from the store |
|  |  | There is no veterinarian/para-veterinarian in my village |
|  |  | There are veterinarians/para-veterinarians but it is hard to seek advice |
|  |  | I do not see the need for seeking advice |
|  |  | Others (Please specify) |
| 49 | How do you ensure the quality of antimicrobials/antibiotics that you buy? (Select all that apply) | I check the expiry date |
|  |  | I make sure I get it from the pharmacy |
|  |  | I go for certain brands |
|  |  | I go for the seller’s recommendations |
|  |  | I go for others’ recommendations (family members, friends, neighbors, etc.) |
|  |  | I do not check |
| 50 | How do you use antimicrobials/antibiotics in your farm? (Select all that apply) | Mixed with feeds |
|  |  | Liquid mix |
|  |  | Injected |
|  |  | Others (Please specify) |
| 51 | When using antimicrobials/antibiotics, how long do you use them in your farm? | Response |
|  |  | Others (Please specify) |
| 52 | What records/information do you keep in managing your farm? (Select all that apply) | Vaccination record |
|  |  | Animal population |
|  |  | Antimicrobials/antibiotics administered/used |
|  |  | Disinfection records |
|  |  | Sales records |
|  |  | Others (Please specify) |
| 53 | Have you used antimicrobials/antibiotics to treat any of the following diseases? (Select all that apply) | Highly Pathogenic Avian Influenza/Avian flu |
|  |  | Foot and mouth disease |
|  |  | Classical swine fever |
|  |  | Newcastle disease |
|  |  | Mastitis |
|  |  | Others (Please specify) |
| 54 | Please describe what you do with excess antimicrobials/antibiotics. (Select all that apply) | Throw in the garbage |
|  |  | Bury in the ground |
|  |  | Burn them |
|  |  | Give to my neighbors/other farmers |
|  |  | Keep them for future use |
|  |  | Others (Please specify) |
| 55 | Please indicate the average time per day that you spend seeking news on your preferred sources of news | Television |
|  |  | Radio |
|  |  | Newspapers/Print media |
|  |  | Internet |
|  |  | Social media |
|  |  | Other (<strong>Please identify and indicate average time spent</strong>) |
| 56 | Please rank your top three most preferred sources of news | Television |
|  |  | Radio |
|  |  | Newspapers/Print media |
|  |  | Internet |
|  |  | Social media |
|  |  | Other (<strong>Please identify and rank</strong>) |
| 57 | Please indicate the average time per day that you spend seeking entertainment on your preferred sources of entertainment | Television |
|  |  | Radio |
|  |  | Newspapers/Print media |
|  |  | Internet |
|  |  | Social media |
|  |  | Other (<strong>Please identify and indicate average time spent</strong>) |
| 58 | Please rank your top three most preferred sources of entertainment | Television |
|  |  | Radio |
|  |  | Newspapers/Print media |
|  |  | Internet |
|  |  | Social media |
|  |  | Other (<strong>Please identify and rank</strong>) |
| 59 | Between news and entertainment, please rate the time you consume on an average day with the far left as mostly news and the far right as mostly entertainment | Open-Ended Response |
| 60 | From which media/informational sources do you usually seek information on animal (terrestrial and aquatic) and plant health issues? (Select all that apply) | Television |
|  |  | Radio |
|  |  | Internet |
|  |  | Social media |
|  |  | Animal/plant health worker |
|  |  | Others (Please specify) |
| 61 | From the following list, rank the top three information sources you find most useful for providing information on animal (terrestrial and aquatic)/plant health and farm issues | Posters |
|  |  | Leaflets |
|  |  | Brochures |
|  |  | Booklets |
|  |  | Village/community billboards |
|  |  | Others (<strong>Please specify and rank</strong>) |
| 62 | How would you like to learn about a new process/method in farming? (Select all that apply) | Drama/Acting/Theatre |
|  |  | Music |
|  |  | Talk show |
|  |  | Practical learning /Training |
|  |  | Advertisement |
|  |  | Others (Please specify) |
| 63 | What language is preferable for antimicrobial/antibiotic resistance information sharing? | Response |

**Animal Health Service Providers Questionnaire**

| Respondent ID |  |
| --- | --- |
| Collector ID |  |
| Start Date |  |
| End Date |  |
| IP Address |  |
| Email Address |  |
| First Name |  |
| Last Name |  |
| Custom Data 1 |  |
| Gender | Response |
| Age | Response |
| What is your highest education level attained? | Response |
|  | Please specify (specialization) |
| How would you classify your profession? (Select all that apply) | Proprietor/business owner |
|  | Company sales representative |
|  | Company management staff |
|  | In-store salesperson/staff |
|  | Veterinary pharmacist (employee) |
|  | Government veterinarian |
|  | Non-government veterinarian |
|  | Para-veterinarian/animal health worker |
|  | Animal health authority (non-veterinarian) |
|  | Other (Please specify) |
| Country of Residence/Nationality | Open-Ended Response |
| Employer/Organization | Open-Ended Response |
| How many years of work experience do you have? | Response |
| Have you encountered or experienced animal disease outbreaks in the last 12 months? | Response |
|  | If yes, please state the encounter |
| Can you describe what antimicrobials are? | Response |
|  | If yes, please describe |
| Can you describe what antibiotics are? | Response |
|  | If yes, please describe |
| Do you think there is a difference between antibiotics and antimicrobials? | Response |
|  | Please explain your response |
| Please list three antimicrobials/antibiotics that you know (generic or brand name) | a |
|  | b |
|  | c |
| All antimicrobials/antibiotics used in humans are also used in animals, and all antimicrobials/antibiotics used in animals are also used in humans | Response |
|  | Other responses |
| Have you heard of antimicrobial resistance or antibiotic resistance? | Response |
|  | If yes, what do you know about it? |
| Where did you hear about antimicrobial/antibiotic resistance? (Select all that apply) | From television |
|  | From radio |
|  | From print media – newspapers, magazines, etc. |
|  | From my friends |
|  | From my colleagues |
|  | From a seminar or workshop |
|  | Social media such as Facebook, Twitter or Instagram |
|  | School or training program |
|  | Other (Please specify) |
| Select what best describes antimicrobial/antibiotic resistance in the list below. (Select all that apply) | Antimicrobial/antibiotic resistance is dangerous but I do not know how to describe it |
|  | When an infection cannot be treated because the medicine is ineffective |
|  | When bacteria develop resistance to one or more antimicrobials/antibiotics |
|  | I am not sure or I do not know |
| Which of the following is true regarding the nature of antimicrobial/antibiotic resistance? (Select all that apply) | Antimicrobial/antibiotic resistance is when infections cannot be treated because medicines lose their potency or effectiveness |
|  | Antimicrobial/antibiotic resistance is when bacteria develop resistance to antimicrobials such as antibiotics |
|  | Antimicrobial/antibiotic resistance emergence is accelerated with overuse and misuse of antimicrobials/antibiotics |
|  | Antimicrobial/antibiotic resistance occurs when microorganisms change in response to the use of antimicrobials/antibiotics |
|  | Antimicrobial/antibiotic resistance traits (genetic elements) can be transferred from one organism to another |
| Which of the following is true about antimicrobial/antibiotic resistance as a health issue? (Select all that apply) | Antimicrobial/antibiotic resistance is a health issue concerning animals (terrestrial and aquatic), plants and humans |
|  | Antimicrobial/antibiotic resistance occurs in human pathogens only |
|  | Antimicrobial/antibiotic resistance in food-producing animals is detected by determining the amount of antimicrobial/antibiotic residue in meat samples |
|  | Antimicrobial/antibiotic resistance is a global health threat |
|  | Antimicrobial/antibiotic resistance can be eradicated |
| Which of the following practices can contribute to the emergence and spread of antimicrobial/antibiotic resistance? (Select all that apply) | Use of antimicrobials/antibiotics in humans when it is not necessary |
|  | Overuse of antimicrobials/antibiotics in animals |
|  | Discontinuing antimicrobial/antibiotic use once the patient (animal or human) shows improvement |
|  | Practicing appropriate use of antimicrobials/antibiotics in animals |
|  | Practicing appropriate use of antimicrobials/antibiotics in humans |
|  | Using fertilizer or water containing animal feces with antimicrobial/antibiotic residues |
| Which of the following practices can help control/prevent the development of antimicrobial/antibiotic resistance? (Select all that apply) | Good vaccination programs |
|  | Good husbandry practices |
|  | Use of antimicrobials/antibiotics as growth promoters |
|  | Good Farm hygiene, sanitation, biosecurity |
|  | Prudent use of antimicrobials/antibiotics |
|  | None of the above |
| Please describe how the use of antimicrobials/antibiotics could affect you and your family | Open-Ended Response |
| How would you rate your situation if one of your family members had an infection that cannot be treated with medicines? | Response |
| Are you concerned you will get an antimicrobial/antibiotic resistance related disease? | Response |
| Are you worried or concerned about antimicrobial/antibiotic resistance issues in the future? | Response |
| Do you think the Government (Ministry of Health & Ministry of Agriculture etc.)/policy makers are worried or concerned about antimicrobial/antibiotic resistance issues in the future? | Response |
| Do you think farmers are worried or concerned about antimicrobial/antibiotic resistance issues in the future? | Response |
| Do you think food consumers are worried or concerned about antimicrobial/antibiotic resistance issues in the future? | Response |
| Please rate your opinion on the following statements whether you Strongly agree; Agree; are Neutral; Disagree; or Strongly disagree: | Antimicrobials such as antibiotics protect both humans and animals from diseases |
|  | It is appropriate to use antimicrobials/antibiotics to improve growth of animals |
|  | There is no danger in giving antimicrobials/antibiotics to humans if properly used when required |
|  | There is no danger in giving antimicrobials/antibiotics to animals if properly used when required |
|  | Antimicrobials/antibiotics should be given with prescription |
|  | It is important to use antimicrobials/antibiotics in farms |
| How would the following factors affect your decision when prescribing or selling antimicrobials/antibiotics? | Opinions of my family members |
|  | News and reports from the media |
|  | Laboratory data |
|  | Opinions of the private industry |
|  | Client/farmer demands |
|  | Opinions of veterinary professional groups |
|  | Data in scientific journals |
|  | Data from online search engines such as Google, Yahoo, Bing |
|  | Potential earnings or financial gain |
|  | Adherence to laws and legislation |
|  | Other factors<strong> (Please identify and indicate level of influence</strong>) |
| Please rate your interest in learning more about antimicrobials/antibiotics | Response |
| Where do you source the antimicrobials/antibiotics you prescribe/sell/administer? (Select all that apply) | Response |
|  | Others (Please specify) |
| What information or guidance do you receive from your antimicrobials/antibiotics suppliers? (Select all that apply) | Response |
|  | Others (Please specify) |
| Would you know if the feed you have bought contains antimicrobials/antibiotics? | Response |
|  | If yes, how would you know? |
| Do you buy feed that already has antimicrobials/antibiotics mixed in and then sell it? | Response |
| Do you mix antimicrobials/antibiotics into the feed that you sell? | Response |
|  | What determines your answer above? |
| If you answered “Always” or “Sometimes” for question 35, what antimicrobials/antibiotics and how much (what concentration or ratio) do you put in the feed? | Open-Ended Response |
| If you answered “Always” or “Sometimes” for question 35, do you switch antimicrobials/antibiotics if the same farmer comes back another time or always mix their feed with the same antimicrobials/antibiotics? | Response |
| Do customers demand for antimicrobials/antibiotics (or feed with antimicrobials/antibiotics)? | Response |
| Do you always demand for a prescription when a farmer asks? | Response |
| If you answered “Sometimes” or “No” for question 39, please tell us why? (Select all that apply) | It is not required by the law |
|  | It is not required by my organization/business |
|  | I do not feel the need |
|  | The clients prefer not to get the prescription |
|  | Others (Please specify) |
| Do you advise clients/customers how to use antimicrobials/antibiotics in farms? | Response |
| If you answered “Sometimes” or “No”, please tell us why? (Select all that apply) | It is not required by the law |
|  | It is not required by my organization/business |
|  | I don’t feel the need |
|  | Others (Please specify) |
| When do you advise clients/customers to use/buy antimicrobials/antibiotics for use in farms? (Select all that apply)﻿ | If they have a prescription from their veterinarian/animal health specialist |
|  | When we have a promotion on antimicrobials/antibiotics |
|  | If the customer asks |
|  | Others (Please specify) |
| How do you advise clients/customers on the use antimicrobials/antibiotics in farms/animals? | Response |
|  | Others (Please specify) |
| Do you keep a record of number of prescriptions you issue to clients/customers? | Response |
| Do you keep records of people bringing in prescriptions? | Response |
| Have you advised the use of antimicrobials/antibiotics to treat any of the following diseases? (Select all that apply) | Highly pathogenic avian influenza/avian flu |
|  | Foot and mouth disease |
|  | Classical swine fever |
|  | Newcastle disease |
|  | Others (Please specify) |
| How often do you meet clients/farmers who use expired/excess antimicrobials/antibiotics? | Response |
| What do you usually advise to clients/farmers when they have excess antimicrobials/antibiotics? (Select all that apply) | Throw in the garbage |
|  | Bury in the ground |
|  | Burn them |
|  | Give to neighbors/other farmers |
|  | Keep them for future use |
|  | Others (Please specify) |
| Do you encounter clients and farmers that have expired antimicrobials/antibiotics? | Response |
|  | If yes how often? |
| What do you usually advise to clients/farmers when they have expired antimicrobials/antibiotics (Select all that apply) | Throw in the garbage |
|  | Bury in the ground |
|  | Burn them |
|  | Give to neighbors/other farmers |
|  | Keep them for future use |
|  | Others (Please specify) |
| Please indicate the average time per day that you spend seeking news on your preferred sources of news | Television |
|  | Radio |
|  | Newspapers/Print media |
|  | Internet |
|  | Social media |
|  | Other (<strong>Please identify and indicate average time spent</strong>) |
| Please rank your top three most preferred sources of news | Television |
|  | Radio |
|  | Newspapers/Print media |
|  | Internet |
|  | Social media |
|  | Other (<strong>Please identify and rank</strong>) |
| Please indicate the average time per day that you spend seeking entertainment on your preferred sources of entertainment | Television |
|  | Radio |
|  | Newspapers/Print media |
|  | Internet |
|  | Social media |
|  | Other (<strong>Please identify and indicate average time spent</strong>) |
| Please rank your top three most preferred sources of entertainment | Television |
|  | Radio |
|  | Newspapers/Print media |
|  | Internet |
|  | Social media |
|  | Other (<strong>Please identify and rank</strong>) |
| Between news and entertainment, please rate the time you consume on an average day with the far left as mostly news and the far right as mostly entertainment | Open-Ended Response |
| From which media/informational sources do you usually seek information on animal health issues? (Select all that apply) | Television |
|  | Radio |
|  | Internet |
|  | Social media |
|  | Animal health worker |
|  | Drug supplier |
|  | Others (Please specify) |
| From the following list, rank the top three information sources you find most useful for providing information on animal health/farm issues | Vet school, symposia, conferences in university |
|  | Online courses |
|  | Websites |
|  | Online blogs |
|  | Social media |
|  | Documentary, television |
|  | Newspapers/Print media |
|  | Posters |
|  | Leaflets |
|  | Brochures |
|  | Booklets |
|  | Village/community billboards |
|  | Drug supplier information materials |
|  | Others (<strong>Please specify and rank</strong>) |
| What language is preferable for antimicrobial/antibiotic resistance information sharing? | Response |

**One Health Practitioners Questionnaire**

| Respondent ID |  |
| --- | --- |
| Collector ID |  |
| Start Date |  |
| End Date |  |
| IP Address |  |
| Email Address |  |
| First Name |  |
| Last Name |  |
| Custom Data 1 |  |
| Gender | Response |
| Age | Response |
| What is your highest education level attained? | Response |
|  | Please specify (specialization) |
| Country of Residence/Nationality | Open-Ended Response |
| Employer/Organization | Open-Ended Response |
| Which sector do you work in? (Select all that apply) | Animal health – Government |
|  | Animal health – International organization, development agencies |
|  | Public/Human health – Government |
|  | Public/Human health - International organization, development agencies |
|  | Environment - Government |
|  | Environment - International organization, development agencies |
|  | Crop production – Government |
|  | Crop production - International organization, development agencies |
|  | Plant health - Government |
|  | Plant health - International organization, development agencies |
|  | Food Safety – Government |
|  | Food Safety – International organization, development agencies |
|  | Regional Economic Community |
|  | Others (Please specify) |
| How many years of work experience do you have? | Response |
| Can you describe what antimicrobials are? | Response |
|  | If yes, please describe |
| Can you describe what antibiotics are? | Response |
|  | If yes, please describe |
| Do you think there is a difference between antibiotics and antimicrobials? | Response |
|  | Please explain your response |
| Please list three antimicrobials/antibiotics that you know (generic or brand name) | a |
|  | b |
|  | c |
| All antimicrobials/antibiotics used in humans are also used in animals, and all antimicrobials/antibiotics used in animals are also used in humans | Response |
|  | Other responses |
| Have you heard of antimicrobial resistance or antibiotic resistance? | Response |
|  | If yes, what do you know about it? |
| Where did you hear about antimicrobial/antibiotic resistance? (Select all that apply) | From television |
|  | From radio |
|  | From print media – newspapers, magazines, etc. |
|  | From my friends |
|  | From my colleagues |
|  | From a seminar or workshop |
|  | Social media such as Facebook, Twitter or Instagram |
|  | School or training program |
|  | Other (Please specify) |
| Select what best describes antimicrobial/antibiotic resistance in the list below. (Select all that apply) | Antimicrobial/antibiotic resistance is dangerous but I do not know how to describe it |
|  | When an infection cannot be treated because the medicine is ineffective |
|  | When bacteria develop resistance to one or more antimicrobials/antibiotics |
|  | I am not sure or I do not know |
| Which of the following is true regarding the nature of antimicrobial/antibiotic resistance? (Select all that apply) | Antimicrobial/antibiotic resistance is when infections cannot be treated because medicines lose their potency or effectiveness |
|  | Antimicrobial/antibiotic resistance is when bacteria develop resistance to antimicrobials such as antibiotics |
|  | Antimicrobial/antibiotic resistance emergence is accelerated with overuse and misuse of antimicrobials/antibiotics |
|  | Antimicrobial/antibiotic resistance occurs when microorganisms change in response to the use of antimicrobials/antibiotics |
|  | Antimicrobial/antibiotic resistance traits (genetic elements) can be transferred from one organism to another |
| Which of the following is true about antimicrobial/antibiotic resistance as a health issue? (Select all that apply) | Antimicrobial/antibiotic resistance is a health issue concerning animals (terrestrial and aquatic), plants and humans |
|  | Antimicrobial/antibiotic resistance occurs in human pathogens only |
|  | Antimicrobial/antibiotic resistance in food-producing animals is detected by determining the amount of antimicrobial/antibiotic residue in meat samples |
|  | Antimicrobial/antibiotic resistance is a global health threat |
|  | Antimicrobial/antibiotic resistance can be eradicated |
| Which of the following practices can contribute to the emergence and spread of antimicrobial/antibiotic resistance? (Select all that apply) | Use of antimicrobials/antibiotics in humans when it is not necessary |
|  | Overuse of antimicrobials/antibiotics in animals |
|  | Discontinuing antimicrobial/antibiotic use once the patient (animal or human) shows improvement |
|  | Practicing appropriate use of antimicrobials/antibiotics in animals |
|  | Practicing appropriate use of antimicrobials/antibiotics in humans |
|  | Using fertilizer or water containing animal feces with antimicrobial/antibiotic residues |
| Which of the following practices can help control/prevent the development of antimicrobial/antibiotic resistance? (Select all that apply) | Good vaccination programs |
|  | Good husbandry practices |
|  | Use of antimicrobials/antibiotics as growth promoters |
|  | Good Farm hygiene, sanitation, biosecurity |
|  | Prudent use of antimicrobials/antibiotics |
|  | None of the above |
| Please describe how the use of antimicrobials/antibiotics could affect you and your family | Open-Ended Response |
| How would you rate your situation if one of your family members had an infection that cannot be treated with medicines? | Response |
| Are you concerned you will get an antimicrobial/antibiotic resistance related disease? | Response |
| Do you think animal feed producers are worried or concerned about antimicrobial/antibiotic resistance issues in the future? | Response |
| Do you think the agency in which you are based in is worried or concerned about antimicrobial/antibiotic resistance issues in the future? | Response |
| Do you think farmers in your region/country are worried or concerned about antimicrobial/antibiotic resistance issues in the future? | Response |
| Do you think food consumers in your region/country are worried or concerned about antimicrobial/antibiotic resistance issues in the future? | Response |
| Please rate your opinion on the following statements whether you Strongly agree; Agree; are Neutral; Disagree; or Strongly disagree: | Antimicrobials such as antibiotics protect both humans and animals from diseases |
|  | It is appropriate to use antimicrobials/antibiotics to improve growth of animals |
|  | There is no danger in giving antimicrobials/antibiotics to humans if properly used when required |
|  | There is no danger in giving antimicrobials/antibiotics to animals if properly used when required |
|  | Antimicrobials/antibiotics should be given with prescription |
|  | It is important to use antimicrobials/antibiotics in farms |
| How would the following factors affect your decision when prioritizing antimicrobial/antibiotic resistance issue in your organization’s agenda? | Opinions of my family members |
|  | News and reports from the media |
|  | Laboratory data |
|  | Opinions of the private industry |
|  | Client/farmer expectations |
|  | Opinions of veterinary professional groups |
|  | Data in scientific journals |
|  | Data from online search engines such as Google, Yahoo, Bing |
|  | Potential earnings or financial gain |
|  | Opinions of senior management in your agency |
|  | Adherence to laws and legislation |
|  | Other factors<strong> (Please identify and indicate level of influence</strong>) |
| Please rate your interest in learning more about antimicrobials/antibiotics | Response |
| Currently, are there any policies or legislations (antimicrobial/antibiotic monitoring and surveillance, drugs sale and use, pharmacovigilance, etc.) to mitigate antimicrobial/antibiotic use or antimicrobial/antibiotic resistance in your region? Do the policies or legislations specifically address antimicrobial/antibiotic resistance within the sector you work in? | Response |
|  | If yes and addressing only a few sectors, please specify the sectors |
| Has your country put in place an implementation or action plan for its antimicrobial/antibiotic resistance strategy and/or policy and legislation? | Response |
|  | If yes, please provide more details and status of implementation |
| Do you have any joint circulars with one health partners to mitigate antimicrobial use and antimicrobial/antibiotic resistance in your region? | Response |
| Do you have any good practices/case studies or/ongoing initiatives related to antimicrobial/antibiotic use or antimicrobial/antibiotic resistance in your country? | Response |
|  | If yes, please add more details including description, activities, donor and web link |
| Is there any committee or working group to discuss antimicrobial/antibiotic resistance issues with the One Health partners? | Response |
| How many times does the committee or working group meet in a year? | Response |
| How often do you meet the One Health partners in both formal and informal settings a year? | Response |
| What kind of antimicrobial/antibiotic resistance support would you like to receive in the future? (Select all that apply) | Coordination with One Health partners & Regional Economic Communities |
|  | Training and capacity building in antimicrobial/antibiotic resistance surveillance and monitoring |
|  | Policy consultation |
|  | Training and capacity building in risk communication |
|  | Pre-made/editable antimicrobial/antibiotic resistance communication materials |
|  | Others (Please specify) |
| Please indicate the average time per day that you spend seeking news on your preferred sources of news | Television |
|  | Radio |
|  | Newspapers/Print media |
|  | Internet |
|  | Social media |
|  | Other (<strong>Please identify and indicate average time spent</strong>) |
| Please rank your top three most preferred sources of news | Television |
|  | Radio |
|  | Newspapers/Print media |
|  | Internet |
|  | Social media |
|  | Other (<strong>Please identify and rank</strong>) |
| Please indicate the average time per day that you spend seeking entertainment on your preferred sources of entertainment | Television |
|  | Radio |
|  | Newspapers/Print media |
|  | Internet |
|  | Social media |
|  | Other (<strong>Please identify and indicate average time spent</strong>) |
| Please rank your top three most preferred sources of entertainment | Television |
|  | Radio |
|  | Newspapers/Print media |
|  | Internet |
|  | Social media |
|  | Other (<strong>Please identify and rank</strong>) |
| Between news and entertainment, please rate the time you consume on an average day with the far left as mostly news and the far right as mostly entertainment | Open-Ended Response |
| From which media/informational sources do you usually seek information on animal/public health issues? (Select all that apply) | Television |
|  | Radio |
|  | Internet |
|  | Social media |
|  | Colleagues, co-workers |
|  | Others (Please specify) |
| From the following list, rank the top three information sources you find most useful for providing information on animal/public health and antimicrobial/antibiotic resistance issues | Symposia and conferences |
|  | Online courses |
|  | Websites and links |
|  | Documentaries |
|  | Television |
|  | Newspapers/Print media |
|  | Posters |
|  | Leaflets |
|  | Brochures |
|  | Online blogs |
|  | Social media |
|  | Others (<strong>Please specify and rank</strong>) |
| What language is preferable for antimicrobial/antibiotic resistance information sharing? | Response |
